# Ciliary Neurotrophic Factor in Skeletal Muscle of Older Adults and the Effects of Weight Loss and Aerobic Training

**DOI:** 10.1093/geroni/igaf122.2733

**Published:** 2025-12-31

**Authors:** Alice Ryan, Guoyan Li, Colleen Lynch, Sausan Jaber, Galya Bigman

**Affiliations:** University of Maryland School of Medicine, Baltimore, Maryland, United States; University of Maryland School of Medicine, Baltimore, Maryland, United States; Baltimore GRECC, Baltimore, Maryland, United States; Baltimore VA Medical Center, Baltimore, Maryland, United States; University of Maryland, Baltimore County, Baltimore, Maryland, United States

## Abstract

Ciliary neurotrophic factor (CNTF), a pluripotent cytokine which acts both centrally and peripherally, has been proposed to alter obesity, and to be modified by aging. There is limited work of CNTF in skeletal muscle in humans and its role in cardiometabolic health. The purpose of this study was to determine skeletal muscle CNTF mRNA expression and its relationship to obesity and glucose tolerance in older adults and to examine the effects of six-month weight loss (WL) and aerobic exercise training (AEX) interventions on its expression. Sixty-seven adults (age 61±3 yr, X±SD) underwent a vastus lateralis skeletal muscle biopsy, VO2max test, DXA scan, and 3-hour oral glucose tolerance test. A subset completed 6 months of WL (n = 18) or 3x/week AEX (n = 16). Skeletal muscle CNTF expression was associated with 3-hr glucose and insulin area under the curve (AUC) (r = 0.287, p = 0.03 and r = 0.289, p = 0.04) but not BMI, %body fat, and VO2max. There was a reduction in body weight (p < 0.001), %fat, glucoseAUC, and insulinAUC (all p < 0.01) after WL. VO2max increased (p < 0.001), %fat decreased (p < 0.05), and body weight, glucoseAUC and insulinAUC did not change after AEX. Muscle CNTF expression decreased after WL (6.16±2.16 vs. 4.81±1.44 AU, p = 0.01) but did not change with AEX (6.90±2.52 vs. 6.65±1.86 AU). CNTF expression in skeletal muscle is related to glucose tolerance but does not appear to be associated with body composition or fitness. Although muscle CNTF mRNA expression is reduced by weight loss, further work is needed to determine its influence on glucose metabolism and obesity in older adults.

